# A Functional Polymorphism (rs2494752) in the *AKT1* Promoter Region and Gastric Adenocarcinoma Risk in an Eastern Chinese Population

**DOI:** 10.1038/srep20008

**Published:** 2016-01-28

**Authors:** Meng-Yun Wang, Jing He, Mei-Ling Zhu, Xiao-Yan Teng, Qiao-Xin Li, Meng-Hong Sun, Xiao-Feng Wang, Ya-Jun Yang, Jiu-Cun Wang, Li Jin, Ya-Nong Wang, Qing-Yi Wei

**Affiliations:** 1 Cancer Institute, Collaborative Innovation Center for Cancer Medicine, Fudan University Shanghai Cancer Center, Shanghai 200032, China; 2 Department of Oncology, Shanghai Medical College, Fudan University, Shanghai 200032, China; 3 Department of Pediatric Surgery, Guangzhou Women and Children’s Medical Center, Guangzhou Medical University, Guangzhou 510623, Guangdong, China; 4 Department of Oncology, Xin Hua Hospital Affiliated To Shanghai Jiao Tong University School of Medicine, Shanghai 200092, China; 5 Department of Pathology, Fudan University Shanghai Cancer Center, Shanghai 200032, China; 6 Ministry of Education Key Laboratory of Contemporary Anthropology, State Key Laboratory of Genetic Engineering, School of Life Sciences, Fudan University, Shanghai 200433, China; 7 Fudan-Taizhou Institute of Health Sciences, Taizhou 225300, Jiangsu, China; 8 Department of Abdominal Surgery, Fudan University Shanghai Cancer Center, Shanghai 200032, China; 9 Duke Cancer Institute, Duke University Medical Center, Durham, NC 27710, USA

## Abstract

AKT is an important signal transduction protein that plays a crucial role in cancer development. Therefore, we evaluated associations between single nucleotide polymorphisms (SNPs) in the *AKT* promoter region and gastric cancer (GCa) risk in a case-control study of 1,110 GCa patients and 1,114 matched cancer-free controls. We genotyped five SNPs (*AKT1* rs2494750G >C, *AKT1* rs2494752A >G, *AKT1* rs10138227C >T, *AKT2* rs7254617G>A and *AKT2* rs2304186G >T) located in the 5′ upstream regulatory, first intron or promoter regions. In the logistic regression analysis, a significantly elevated GCa risk was associated with the rs2494752 AG/GG variant genotypes (adjusted odds ratio [OR] = 1.20, 95% confidence interval [CI] = 1.02–1.42) under a dominant genetic model, and this risk was more evident in subgroups of ever drinkers. The luciferase reporter assay showed that the rs2494752 G allele significantly increased luciferase activity. Our results suggest that the potentially functional *AKT1* rs2494752 SNP may affect GCa susceptibility, likely by modulating the *AKT1* promoter transcriptional activity. Larger, independent studies are warranted to validate our findings.

Gastric cancer is the second leading cause of cancer-related death and the fourth most common cancer world-wide, with an estimated 951,600 new cases and 723,100 deaths in 2012^1^. Over 70% of new cases and deaths occur in developing countries with the highest incidence rates in Eastern Asia, Eastern Europe, and South America^1^. Up to now, numerous studies have revealed that gastric cancer is a complex disease likely caused by the *H. pylori* infection, environmental exposures and genetic factors in a multi-step process of carcinogenesis.

AKT (also known as protein kinase B or PKB), a 57 kD serine-threonine kinase, is one of the key molecules that activate downstream of the PI3 kinase signaling pathway^2^. The AKT family comprises three closely and evolutionary related isoforms (AKT1, AKT2 and AKT3 or PKBα, PKBβ and PKBγ), and each AKT member contains an N-terminal pleckstrin homology (PH) domain, a central kinase domain and a C-terminal regulatory domain^3^. The chromosomal location of each human *AKT* gene has been identified as 14q32 (*AKT1*), 19q13.1–13.2 (*AKT2*), and 1q44 (*AKT3*) by fluorescence *in situ* hybridization^4^,^5^,^6^. AKT phosphorylates and/or their interactions with a number of molecules regulate many cellular processes, including metabolism, proliferation, cell survival, growth and angiogenesis^7^. Over-activation of AKT can influence many downstream effectors and multiple pathways that favor tumorigenesis; therefore, AKT is one of the most frequently hyperactivated protein kinases in human cancer^8^.

A number of studies have reported amplification of the *AKT* genes in a variety of human cancers. For example, Staal *et al.* detected *AKT1* amplification in a single gastric carcinoma, and *AKT1* was originally identified as a potential human oncogene^4^. Other studies have also found *AKT1* amplification in glioblastoma^9^. *AKT2* amplification has been reported in cancers of the ovary, pancreas, stomach, and breast^10^,^11^. *AKT2* amplification was particularly associated with high-grade aggressive ovarian cancers and appeared to occur as part of the frequent amplification of the 19q13.1–q13.2 chromosomal region^12^. Over-expression of *AKT3* mRNA was detected in breast and prostate cancers^6^. In addition, somatic mutations, such as those in *AKT1* E17K, have been identified in breast, colorectal and ovarian cancers^13^. However, mutations in *AKT* itself are extremely rare, and dysregulation of the pathway possibly results from mutations or altered expression of an upstream regulator of the AKT activity^14^.

Numerous studies have found that single nucleotide polymorphisms (SNPs) in many genes, often in signal transduction pathways, contribute to the origin, propagation, and treatment responses of a cancer^15^. One study reported that SNPs in *AKT1* and *AKT2* were associated with recurrence, survival, and responsiveness to chemotherapy in esophageal cancer^16^. In a study of gastric cancer, Wang *et al.*^17^ investigated six selected SNPs located in the AKT pathway genes and found that the *AKT1* rs2498804 GG genotype was associated with lower AKT1 activation in gastric cancer tissues, and consequentially the recurrence rate was reduced by 30.4%, and the survival rate was increased by 33.7% in patients who carried the *AKT1* rs2498804 GG genotype. In light of the critical role of the *AKT* pathway in maintaining proper cellular function, it is possible that some functional SNPs of genes involved in this pathway may have an effect on cancer risk.

Previous pre-GWAS studies have investigated associations between genetic variations in *AKT* and cancer risk, but the results were inconsistent. For example, Chen *et al.* investigated eight SNPs in the PTEN/AKT/mTOR axis in a case-control study that consisted of 666 prostate cancer patients and 708 cancer-free controls in a Chinese population, and they observed significant associations between prostate cancer risk and *AKT2* rs7254617 variant genotypes^18^. In another study conducted by Wang *et al.*, the *AKT1* rs2498801 homozygous variant genotype was found to be associated with significantly increased risk of endometrial cancer in a recessive genetic model^19^. Sung *et al.* examined six SNPs in the promoter regions of *AKT2* and *AKT3* in 360 lung cancer patients and 360 normal controls and found that the variant genotypes and haplotypes were not significantly associated with risk of lung cancer in a Korean population^20^.

It has been hypothesized that genetic variation within a gene promoter regulatory region might affect gene expression levels, thereby modifying the disease susceptibility. To date, associations between *AKT1* and *AKT2* SNPs and risk of gastric cancer have not yet been clarified. Therefore, we conducted a relatively large hospital-based case-control study of 1,100 gastric cancer patients and 1,144 cancer-free controls in an Eastern Chinese Population to evaluate associations between SNPs in the 5′ upstream regulatory, first intron or promoter regions of *AKT1* and *AKT2* and gastric cancer risk and performed additional experiments to unravel the underlying molecular mechanisms.

## Results

### Characteristics of the Study Population

As shown in Table 1, this study included 1,144 cancer-free controls and 1,100 gastric cancer cases. The cases and controls appeared to be adequately frequency-matched for age and sex (*P* = 0.746 and 0.639, respectively). Compared with the cases, the controls were more likely to be smokers and drinkers (*P* < 0.0001 and *P* = 0.003, respectively). Thereafter, these variables (i.e., age, sex, smoking status and drinking status) were further adjusted for in the subsequent multivariate logistic regression analyses.

### Associations of *AKT1* and *AKT2* genotypes with risk of gastric cancer

The genotype distributions of the five selected SNPs among the cases and controls are summarized in Table 2. The genotype frequencies among the controls were in agreement with the Hardy-Weinberg equilibrium (all *P* > 0.05). Under the dominant genetic model, significantly higher risk of gastric cancer was presented in individuals with rs2494752 variant AG/GG genotypes (adjusted odds ratio [OR] = 1.20, 95% confident interval [CI] = 1.02–1.42), compared with the wild-type homozygous AA genotype carriers. However, no variant genotypes of other four SNPs (rs2494750 CG/CC, rs10138227 CT/TT, rs7254617 AG/GG, and rs2304186 GT/TT) were associated with risk of gastric cancer, compared with their common homozygous genotypes.

### Stratification Analysis

We further performed a stratification analysis of the associations of rs2494752 A >G with risk of gastric cancer by subgroups of age, sex, smoking and drinking status, assuming a dominant genetic model. As shown in Table 3, the stratification analysis indicated that the risk effect of rs2494752 AG/GG genotypes, compared with the AA genotype, was more evident in ever-drinkers (adjusted OR = 1.46, 95% CI = 1.05–2.02). However, homogeneity tests suggested that there was no difference in risk estimates between strata, nor any evidence of gene-environment interactions between the variant genotypes and other selected variables on risk of gastric cancer, nor any differential risk by the site and stage of the tumors (data not shown).

### Association of high-order interactions with gastric cancer risk by multifactor dimensionality reduction (MDR) analysis

To further explore high-order interactions, we performed the MDR analysis by including the five SNPs (i.e., rs2494750 CG/CC, rs2494752 AG/GG, rs10138227 CT/TT, rs7254617 AG/GG and rs2304186 GT/TT) and four risk factors (Supplemental Table 1). The model with the lowest prediction error and the highest cross-validation consistency was selected for different number of factors considered. The reported cross-validation consistency is the number of cross-validation intervals (maximum of 100) that a particular combination was chosen by MDR averaged across the 100 runs. The model that minimized prediction error and maximized the cross-validation consistency was the nine-factor model that included all the five SNPs and four environmental factors, which yielded the minimal average prediction error of 0.383 and the maximal CVC of 100/100. It is worth noticing that as the model size decreases, the prediction error increases, leading to a worse predicting.

Finally, the FPRP values for all significant findings were calculated at different prior probability levels. As shown in Table 4, for a prior probability of 0.1, assuming the OR for specific genotype was 1.50, the FPRP value was 0.184 for a risk association with rs2494752 AG/GG genotype in all individuals. All these significant associations tested by false-positive reporting probability (FPRP) were considered noteworthy, using the criteria of the probability of a false-positive result less than 20%. In contrast, those findings with greater FPRP values may be false positive.

### Effect of the rs2494752 polymorphism on transcriptional activity

**Figure 1:**
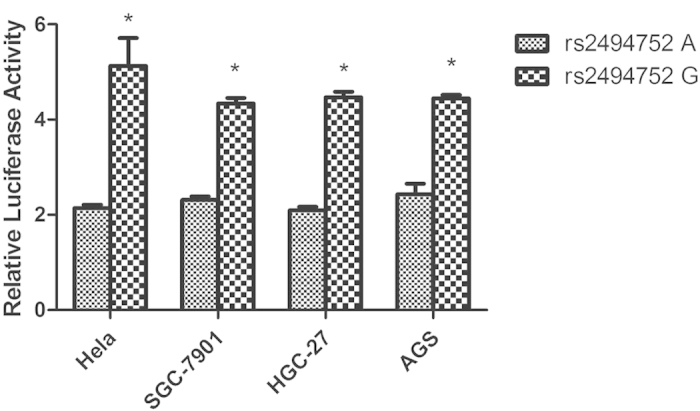
Effect of the *AKT1* rs2494752 polymorphism on the AKT1 promoter activity. (**A**) Schematic representation of reporter plasmids containing the *AKT1* rs2494752 A or G allele, which was inserted at upstream of the luciferase reporter gene in the pGL3-Basic plasmid. (**B**) The two constructs were transiently transfected into the Hela, SGC-7901, HGC-27 and AGS cells, respectively. All of the constructs were cotransfected with pRL-SV40 to standardize the transfection efficiency. Values were means ± SD from more than 3 separate experiments that were each performed in triplicate.

To further evaluate the biological functional effect of rs2494752 SNP on the *AKT1* transcription, we generated reporter gene constructs containing either rs2494752 A or G allele, which were transfected into SGC-7901, HGC-27, AGS and HeLa cell lines with the reporter plasmids. As shown in Fig. 1, the construct containing the rs2494752 G allele showed a significantly higher reporter gene expression, compared with that containing the rs2494752 A allele in these cell lines. These results suggest that the rs2494752 A→G allele change in the promoter region may increase transcriptional activity of the *AKT1*, a possible molecular mechanism underlying the observed associations.

## Discussion

The present study evaluated the effect of five selected potentially functional SNPs of *AKT1* and *AKT2* on risk of gastric cancer. The major finding was a significant association of rs2494752 G variant genotypes in *AKT1* with an elevated gastric cancer risk under a dominant genetic model. The underlying mechanism for the observed association of cancer risk associated with rs2494752 SNP was that the rs2494752 G allele significantly increased the transcriptional activity of *AKT1*.

AKT is activated in cells exposed to all known oncogenic growth factors, angiogenic factors and cytokines by binding to cognate receptors on cell surface. Further, AKT is also activated by constitutively active Ras and Src as well as steroid hormones, such as estrogen and androgen through a mechanism independent of their nuclear receptors^21^,^22^. Constitutive activation of AKT is believed to play an important role in a variety of cellular processors, including metabolism, proliferation and survival, and thus, dysregulation of AKT is associated with several human diseases, including cancer^14^. Overexpression of AKT isoforms has been reported in cancers of the breast, colon, ovary, pancreas, prostate and stomach^23^,^24^,^25^,^26^. Furthermore, immunohistochemical studies using specific antibodies against serine 473-phosphorylated AKT have shown that the AKT activity is detectable in various cancers, including melanoma and cancers of the head and neck, ovary, pancreas, stomach and prostate^25^,^27^,^28^,^29^,^30^,^31^.

The rs2494752 SNP is located within the 5′ near gene region of *AKT1*, a region predicted to be the potential promoter region of *AKT1* based on sequence alignments using the UCSC Genome Browser (http://genome.ucsc.edu), which may disrupt a potential cis-regulatory module affecting gene transcription and translation. Considering the role of AKT1 in facilitating cancer development and progression, the increased levels of AKT1 in the presence of the rs2494752 G variant allele in the promoter may increase cancer susceptibility, which may explain our findings of cancer risk associated with this SNP. It is also possible that this SNP is in linkage disequilibrium (LD) with the real causal SNP located in the coding region and affect the protein function at the posttranslational level. Thus, the sequencing of this gene will be necessary to identify additional causal variants in the future.

Cancer, as a complex disease involving joint effects of multiple genetic variants and environment factors, may arise from the gene-environment interaction. In the present study, our MDR analysis suggested that the overall best MDR model was the nine-factor model that included all the five SNPs and four exposure variables, indicating potential gene-environment interactions. But in the logistic regression analysis for detecting multiplicative interactions among these five SNPs and exposures, no statistical evidence was found for such interactions, which may be due to the relative small effect size of selected SNPs investigated in the present study.

In the stratification analysis, we found that the risk effect of rs2494752 AG/GG variant genotypes was more obvious in subgroups of ever-drinkers. Previous studies showed inconsistent results about alcohol consumption and risk of gastric cancer. A recent meta-analysis found a positive association between heavy alcohol drinking and gastric cancer risk, which may support our findings. However, considering the limited small size in each stratum, we cannot rule out the possibility that these observed results may be by chance. Our results should be interpreted with caution.

In summary, the present hospital-based case-control study investigated associations between five selected potentially functional *AKT1*/*AKT2* SNPs and gastric cancer risk. The strengths include a relatively large sample size from a single institution and diverse approaches to analyze the data, including logistic regression and MDR for accessing possible gene-gene and gene-environmental interactions. Furthermore, the results of laboratory experiments for the functional analysis using plasmid construction provided additional evidence for biological plausibility of the observed associations. However, several methodological issues and limitations of the present study should also be noted. First, there may be selection bias and recall bias by the design of a retrospective study, which may have been minimized by frequency-matching cases and controls as well as the adjustment for potential confounding factors in further multivariate analyses. Second, only five potential functional SNPs of *AKT1*/*AKT2* were investigated in the present study, which did not cover all functional SNPs and may have missed some important variants within the two genes. Third, we did not have the information on Lauren-type such as intestinal type and diffuse type, and we did not have the opportunity to evaluate the role played by genetic factors in the tumor-type specific etiology. Fourth, we were not able to measure the expression of AKT1 and AKT2 in mRNA and protein levels using real-time PCR, Western blot, and immunohistochemistry due to the lack of clinical tissues or samples from the study patient population. Finally, other risk factors, especially the H. pylori infection status, were not available for further analysis due to the nature of the retrospective study design. Therefore, additional large and prospective studies, with carefully collection of detailed clinical characteristics of the patients as well as tumor tissues, are warranted to further confirm our findings.

## Methods

### Study population

This study comprised 1,100 patients with newly diagnosed and histopathologically confirmed primary gastric adenocarcinoma and 1,144 cancer-free controls. All patients were recruited from Fudan University Shanghai Cancer Center (FUSCC) between January 2009 and March 2011. All patients came from Eastern China, including Shanghai City, Jiangsu Province, Zhejiang Province and the surrounding regions. The cancer-free controls were frequency-matched to these cases on sex and age (±5 years) and were recruited by Taizhou Longitudinal Study (TZL) at the same time period in Eastern China as described previously^32^. At recruitment, a written informed consent was obtained from each subject before the in-person interview to obtain demographic data and clinical information, including age, sex, ethnicity, smoking, alcohol consumption and TNM stage. After interview, each participant donated a sample of approximately 10-mL blood, of which 1 mL was used for genomic DNA extraction. This research protocol was approved by the Institutional Review Board of FUSCC, and the experiments on human samples were performed in accordance with relevant guidelines and regulations.

### SNP selection and genotyping

**Figure 2:**
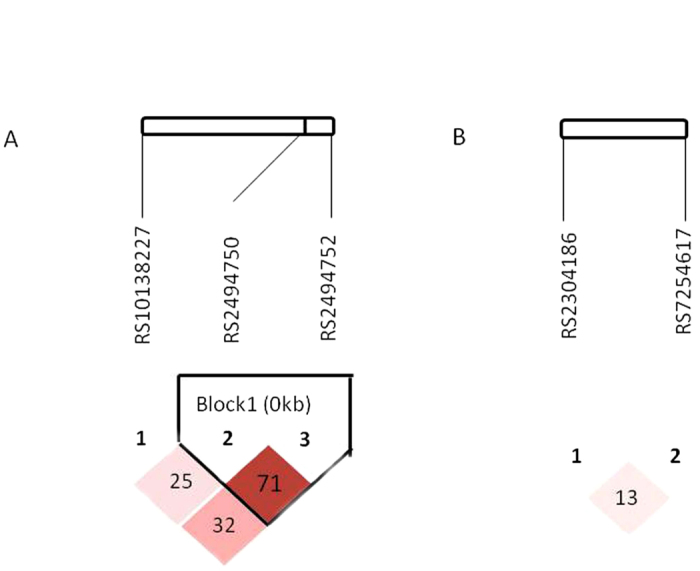
Linkage disequilibrium (LD) blocks of the *AKT1*/*AKT2* genes. (**A**) Pairwise LD among three selected *AKT1* SNPs. The value within each diamond represents the pairwise correlation between SNPs (measured as r^2^) defined by the upper left and the upper right sides of the diamond. The red-to-white gradient reflects higher to lower LD values. (**B**) Pairwise LD among two selected SNPs of *AKT2*.

SNPs of interest for *AKT1 and AKT2* were selected based on the NCBI dbSNP database (http://www.ncbi.nlm.nih.gov/projects/SNP) and SNPinfo (http://snpinfo.niehs.nih.gov/snpfunc.htm). The potentially functional polymorphisms were identified according to the following criteria: (1) the minor allele frequency (MAF) reported in HapMap was >5% in Chinese population; (2) predicted to affect the activity of transcription factor binding site (TFBS) in the 5′ upstream regulatory, first intron or promoter region; (3) with low linkage disequilibrium (LD) using an r^2^ threshold of <0.8 for each paired SNPs (Fig. 2) and (4) not included in the published genome-wide association studies (GWASs) and our previous studies. As a result, five potentially functional SNPs were selected for genotyping: (*AKT1* rs2494750G >C, *AKT1* rs2494752A >G, *AKT1* rs10138227C >T, *AKT2* 7254617G >A and *AKT2* rs2304186G >T).

All genomic DNA was extracted from the peripheral blood using QIAamp DNA Blood mini kit (QIAGEN Inc, Valencia, CA). Genotyping was performed using an Applied Biosystems TaqMan assay on the ABI 7900 HT sequence detection system (Applied Biosystems, Foster City, CA) as previously described^33^. More than 5% of the samples were re-genotyped for each polymorphism, and the results were 100% concordant.

### Construction of promoter-reporter plasmids

To construct the target *AKT1* promoter-reporter plasmids, we synthesized the DNA fragment containing either the rs2494752 A or G allele by amplifying the 1402-bp (from −1991 to −589 base relative to the transcription start site) *AKT1* promoter region using primers with restriction sites. The primers were 5′- CGGGGTACCCCGTCGGGCCCTTCCCTGGTGCAG-3′ (forward) and 5′- CCGCTCGAGTGGGAGAAGAGGGGCACACCTTGC-3′ (reverse), including the *Kpn*I and *Xho*I restriction sites. The resulting PCR products were subsequently digested with *Kpn*I and *Xho*I and cloned into the pGL3-basic vector (Promega, Madison, WI, USA). The constructs were all confirmed by DNA sequencing (data not shown).

### Cell Culture and Treatments

Four cancer cell lines, i.e., the cervical adenocarcinoma HeLa and three gastric cancer cell lines, including SGC-7901, HGC-27 and AGS, were used for the luciferase reporter assay. All these four cell lines were obtained from the American Type Culture Collection (ATCC). SGC-7901, HGC-27 and AGS cell lines were grown in RPMI-1640 medium supplemented with 10% Fetal Bovine Serum (FBS) and HeLa was cultured in Dulbecco’s modified Eagle’s medium with 10% FBS.

### Plasmid Transfection and Dual Luciferase Reporter Assay

SGC-7901, HGC-27, AGS and HeLa cells were seeded in 24-well culture plates. After 24 h, each well was transfected by Lipofectamine 2000 (Invitrogen, Carlsbad, CA, USA) with 0.8 μg of each constructed vector, either with the A or G allele. Simultaneously, all plasmids were cotransfected with 10 ng pRL-SV40 plasmids (Promega) per well, which contained the Renilla luciferase gene as an internal control for correcting transfection efficiency. The pGL3-Basic vector without an insert was used as a negative control. Forty-eight hours after transfections, cells were lysed with the passive lysis buffer (Promega) and assayed for luciferase activity using the Dual-Luciferase Reporter Assay System (Promega). Three independent transfection experiments were performed, and each luciferase assay was carried out in triplicate.

### Statistical analysis

Differences in the distributions of demographic characteristics, selected variables, and frequencies of genotypes between cases and controls were tested by the Student’s *t* test (for continuous variables) or Chi-square-test (for categorical variables). Hardy-Weinberg equilibrium was evaluated by a goodness-of-fit Chi-square test to compare the observed genotype frequencies with the expected among the controls. Univariate and multivariate logistic regression models were used to evaluate the associations between genotypes and risk of gastric cancer by ORs and their 95% CIs with the adjustment for possible confounders. Risk genotypes of studied SNPs were combined to create a genetic score of the number of the observed risk genotypes, and this score was used for further analyses. The MDR software (version 2.0 beta 8.2) was applied to identify possible high-order interactions associated with gastric cancer risk^34^. The FPRP was calculated for all significant results observed in the present study to detect the false-positive findings^35^. We assigned a prior probability of 0.1 to detect an OR of 1.56 (for a risk effect) or 0.67 (for a protective effect) for an association with genotypes of each SNP under investigation. Only significant results with an FPRP value less than 0.20 were considered a noteworthy association. All statistical analyses were performed by using Statistical Analysis Software (v.9.1 SAS Institute, Cary, NC), and all *P* values were two-sided with a 0.05 significance level.

## Additional Information

**How to cite this article**: Wang, M.-Y. *et al.* A Functional Polymorphism (rs2494752) in the *AKT1* Promoter Region and Gastric Adenocarcinoma Risk in an Eastern Chinese Population. *Sci. Rep.* **6**, 20008; doi: 10.1038/srep20008 (2016).

## Supplementary Material

supplementary 1

## Figures and Tables

**Table 1:** Frequency distributions of selected characteristics of gastric cancer cases and cancer-free controls.

Variables	Cases No. (%)	Controls No. (%)	*P*^a^
All subjects	1,100 (100.0)	1,144 (100.0)	
Age, yr			0.746
Range, yr	21–86	22–86	
Mean^b^, yr	58.6 ± 11.37	59.1 ± 11.44	
Age group			
≤50	228 (20.7)	240 (21.0)	
51–60	376 (34.2)	376 (32.9)	
61–70	331 (30.1)	366 (32.0)	
>70	165 (15.0)	162 (14.2)	
Sex			0.639
Males	783 (71.2)	804 (70.3)	
Females	317 (28.8)	340 (29.7)	
Drinking status			0.003
Ever	261 (23.7)	336 (29.4)	
Never	839 (76.3)	808 (70.6)	
Smoking status			<0.0001
Ever	429 (39.0)	572 (50.0)	
Never	671 (61.0)	572(50.0)	
Pack-years			<0.0001
0	671 (61.0)	572 (50.0)	
≤25 (mean)	223 (20.3)	343 (30.0)	
>25 (mean)	206 (18.7)	229 (20.0)	
Tumor site			
Gastroesophageal junction	301 (27.4)	—	
Non-junctional	799 (72.6)	—	
Stage			
I	237 (21.6)	—	
II	224 (20.4)	—	
III	448 (40.7)	—	
IV	191 (17.4)	—	
I + II	461 (41.9)	—	
III + IV	639 (58.1)	—	

^a^Two-sided Chi square test for distributions between cases and controls.

^b^Data were presented as mean ± SD.

**Table 2:** Logistic regression analysis of associations between the genotypes of *AKT1/ AKT2* and gastric cancer risk.

Variants	Genotypes	Cases No. (%)	ControlsNo. (%)	*P*^a^	Crude OR (95% CI)	*P*	Adjusted OR (95% CI)	*P*^b^
Total		1100 (100.0)	1144 (100.00 %)					
*AKT1* rs2494750								
	GG	493 (44.9)	545 (47.6)	0.113^c^	1.00		1.00	0.106^c^
	GC	480 (43.7)	487 (42.6)		1.09 (0.91–1.30)	0.349	1.09 (0.91–1.30)	0.339
	CC	126 (11.5)	112 (9.8)		1.24 (0.94–1.65)	0.134	1.25 (0.94–1.66)	0.126
	CG/CC	606 (55.1)	599 (52.4)		1.12 (0.95–1.32)	0.187	1.12 (0.95–1.33)	0.178
*AKT1* rs2494752								
	AA	547 (49.7)	623 (54.5)	**0.034**^c^	1.00		1.00	**0.040**^c^
	AG	454 (41.3)	430 (37.6)		**1.20 (1.01**–**1.43)**	**0.039**	**1.20 (1.00**–**1.43)**	**0.046**
	GG	99 (9.0)	91 (8.0)		1.24 (0.91–1.68)	0.171	1.23 (0.91–1.68)	0.185
	AG/GG	553 (50.3)	521 (45.5)		**1.21 (1.02**–**1.43)**	**0.025**	**1.20 (1.02**–**1.42)**	**0.030**
*AKT1* rs10138227								
	CC	862 (78.4)	916 (80.1)	0.279^c^	1.00		1.00	0.333^c^
	CT	223 (20.3)	216 (18.9)		1.10 (0.89–1.35)	0.385	1.09 (0.88–1.35)	0.421
	TT	15 (1.4)	12 (1.1)		1.33 (0.62–2.85)	0.468	1.26 (0.59–2.73)	0.551
	CT/TT	238 (21.6)	228 (19.9)		1.11 (0.91–1.36)	0.319	1.10 (0.90–1.35)	0.365
*AKT2* rs7254617								
	GG	823 (74.8)	863 (75.4)	0.834^c^	1.00		1.00	0.693^c^
	AG	255 (23.2)	256 (22.4)		1.05 (0.86–1.27)	0.666	1.08 (0.88–1.31)	0.480
	AA	22 (2.0)	25 (2.2)		0.92 (0.52–1.65)	0.787	0.90 (0.50–1.62)	0.729
	AG/GG	277 (325.2)	281 (24.6)		1.03 (0.85–1.25)	0.735	1.06 (0.87–1.28)	0.562
*AKT2* rs2304186								
	GG	323 (29.4)	340 (29.7)	0.851^c^	1.00		1.00	0.906^c^
	GT	544 (49.5)	564 (49.3)		1.02 (0.84–1.23)	0.877	1.01 (0.83–1.23)	0.908
	TT	233 (21.2)	240 (21.0)		1.02 (0.81–1.29)	0.857	1.01 (0.80–1.29)	0.911
	GT/TT	777 (70.6)	804 (70.3)		1.02 (0.85–1.22)	0.853	1.01 (0.84–1.22)	0.897

CI, confidence interval; OR, odds ratio.

The results were in bold, if the 95% CI excluded 1 and *P* < 0.05.

^a^Chi square test for genotype distributions between cases and controls.

^b^Adjusted for age, sex, smoking and drinking status in logistic regress models.

^c^For additive genetic models.

**Table 3:** Stratification analysis for associations between variant genotypes and gastric cancer risk.

Variables	rs2494752		Crude OR (95% CI)	*P*	Adjusted OR (95% CI)	*P*^a^	*P*_*hom*_
	(cases/controls)						
	AA	AG/GG					
Age							
≤59	290/315	277/257	1.17 (0.93–1.48)	0.185	1.17 (0.92–1.47)	0.195	0.689
>59	257/308	276/264	1.25 (0.99–1.59)	0.062	1.26 (0.99–1.61)	0.058	
Sex							
Females	156/182	161/158	1.19 (0.88–1.62)	0.269	1.21 (0.89–1.65)	0.226	0.896
Males	391/441	392/363	1.22(1.00–1.48)	0.050	1.20 (0.99–1.47)	0.067	
Smoking status							
Never	331/314	340/258	1.25 (1.00–1.56)	0.050	1.25(1.00–1.56)	0.055	0.657
Ever	216/309	213/263	1.16 (0.90–1.49)	0.250	1.17(0.91–1.50)	0.236	
Drinking status							
Never	417/425	422/383	1.12 (0.93–1.36)	0.240	1.13 (0.93–1.37)	0.233	0.191
Ever	130/198	131/138	**1.45 (1.04**–**2.00)**	**0.027**	**1.46 (1.05**–**2.02)**	**0.024**	

CI, confidence interval; OR, odds ratio. *P*_hom_ derived from the homogeneity test.

The results were in bold, if the 95% CI excluded 1 and *P* < 0.05.

^a^Obtained in logistic regression models with adjustment for age, sex, smoking status and drinking status.

**Table 4:** False-positive report probability values for associations between risk of gastric cancer and frequency of genotypes of the *AKT1, AKT2* gene.

Genotype/Haplotype	Crude OR^a^ (95% CI)	*P*^b^	Statistical power^c^	Prior probability				
				0.25	0.1	0.01	0.001	0.0001
*AKT1* rs2494752								
AG vs. AA	1.20 (1.01–1.43)	0.039	0.996	0.105	0.261	0.795	0.975	0.997
AG/GG vs. AA	1.21 (1.02–1.43)	0.025	0.995	0.070	**0.184**	0.713	0.962	0.996
rs2494752 AG/GG vs. AA								
Ever drinker	1.45 (1.04–2.00)	0.027	0.592	0.12	0.291	0.819	0.979	0.998

^a^The crude OR reported in Table 2.

^b^The chi-square test of the genotype distributions reported in Table 2.

^c^Statistical power was calculated using the number of observations in the study and the OR and *P* values in this table.
