# Supplementary material for: A Functional Polymorphism (rs2494752) in the AKT1 Promoter Region and Gastric Adenocarcinoma Risk in an Eastern Chinese Population
Source: Sci Rep. 2016 Jan 28;6:20008. doi: 10.1038/srep20008 (PMC4730221; doi:10.1038/srep20008)
Supplement: supplementary 1 [file srep20008-s1.pdf]

A Functional Polymorphism (rs2494752) in the *AKT1* Promoter Region and Gastric Adenocarcinoma Risk in an Eastern Chinese Population

Meng-Yun Wang[1,2], Jing He[1,3,10], Mei-Ling Zhu[1,4], Xiao-Yan Teng[1,2], Qiao-Xin Li[1,2,] Meng-Hong Sun[5], Xiao-Feng Wang[6,7], Ya-Jun Yang[6,7], Jiu-Cun Wang[6,7], Li Jin[6,7] , Ya-Nong Wang[8], Qing-Yi Wei[1,9*]

1 Cancer Institute, Collaborative Innovation Center for Cancer Medicine, Fudan University Shanghai Cancer Center, Shanghai 200032, China

2 Department of Oncology, Shanghai Medical College, Fudan University, Shanghai 200032, China

3 Department of Pediatric Surgery, Guangzhou Women and Children's Medical Center, Guangzhou Medical University, Guangzhou 510623, Guangdong, China

4 Department of Oncology, Xin Hua Hospital Affiliated To Shanghai Jiao Tong University School of Medicine, Shanghai 200092, China

5 Department of Pathology, Fudan University Shanghai Cancer Center, Shanghai 200032, China

6 Ministry of Education Key Laboratory of Contemporary Anthropology, State Key Laboratory of Genetic Engineering, School of Life Sciences, Fudan University, Shanghai 200433, China

7 Fudan-Taizhou Institute of Health Sciences, Taizhou 225300, Jiangsu, China

8 Department of Abdominal Surgery, Fudan University Shanghai Cancer Center, Shanghai 200032, China

9 Duke Cancer Institute, Duke University Medical Center, Durham, NC 27710, USA

10 These authors contributed equally to this work

Supplemental Table 1. MDR analysis for the risk of gastric cancer prediction with and without *AKT1*, *AKT2* genotypes

Best interaction models	Cross-validation	Average prediction error	*P* [a]
1	100/100	44.6%	<.0001
1,2	64/100	44.5%	<.0001
1,3,4	100/100	43.6%	<.0001
1,4,5,6	63/100	43.0%	<.0001
1,3,4,5,6	97/100	42.5%	<.0001
1,2,3,4,6,7	97/100	41.7%	<.0001
1,2,4, 6,7,8,9	47/100	40.7%	<.0001
1,2,4,5,6,7,8,9	88/100	39.1%	<.0001
**1,2,3,4,5,6,7,8,9**	**100/100**	**38.3%**	**<.0001**

MDR, multifactor dimensionality reduction

[a] *P* value for 1000-fold permutation test

The best model with maximum cross-validation consistency and minimum prediction error rate was in bold

Labels: 1, smoking status; 2, rs2304186; 3, rs2494750; 4, rs2494752; 5,gender; 6. drink status 7, rs7254617; 8, age; 9, rs10138277.
